# Biotransformation of Tropical Fruit By-Products for the Development of Kombucha Analogues with Antioxidant Potential

**DOI:** 10.17113/ftb.62.03.24.8350

**Published:** 2024-09

**Authors:** Gabriel Barbosa Câmara, Giovana Matias do Prado, Paulo Henrique Machado de Sousa, Vanessa Bordin Viera, Helvia Waleska Casullo de Araújo, Amélia Ruth Nascimento Lima, Antonio Augusto Lima Araujo Filho, Ícaro Gusmão Pinto Vieira, Victor Borges Fernandes, Liandra De Souza Oliveira, Larissa Morais Ribeiro da Silva

**Affiliations:** 1 Federal University of Ceará, Campus do Pici, Ac. Público, 856 - Pici, 60020-181 Fortaleza-CE, Brazil; 2 Federal University of Campina Grande, Sítio Olho D'água da Bica - Zona Rural, 58175-000, Cuité-PB, Brazil; 3 State University of Paraiba, R. Baraúnas, 351 - Universitário, 58429-500, Campina Grande-PB, Brazil; 4 PADETEC – Technological Development Park, Federal University of Ceara, Campus do Pici - Pici, 60440-690, Fortaleza - CE, Brazil; 5 University Center Faculty of Medical Sciences in Campina Grande, R. Manoel Cardoso Palhano, 124-152 - Itararé, 58408-326, Campina Grande-PB, Brazil

**Keywords:** fruit by-products, fermented beverages, functional food, innovative food production

## Abstract

**Research background:**

In a country where millions of people have nutritional needs, innovative ways of producing food from commonly wasted agro-industrial by-products, can be an important alternative for the production of fermented beverages. In light of this, the aim of this study is to evaluate the potential of fruit by-products from acerola, guava and tamarind for the production of fermented beverages.

**Experimental approach:**

Physicochemical and microbiological parameters, total antioxidant capacity and fermentation kinetics were investigated during the first (at 0, 48, 72, 96 and 168 h) and second fermentation (at 0 and 24 h). The acid profile of fermented beverages was determined by chromatography, and the sensory profile was determined by consumer acceptance test.

**Results and conclusions:**

Physicochemical parameters of all formulations complied with current legislation and were of satisfactory microbiological quality. The reslts of fermentation kinetics showed that both pH and soluble solids content decreased - with an average final pH of 3.12, 2.85 and 2.78 for the acerola, guava and tamarind formulations, respectively – while acidity increased with final values of 0.94, 0.75 and 1 % for the same formulations. Of all formulations, tamarind had the highest total soluble solids content (8.17 g/100 g), and acerola had the highest antioxidant potential determined as Trolox equivalents ((20.0±0.8) μM/g). Organic acids were found in all samples, with mainly glucuronic acid detected in the kombucha beverages. All formulations showed satisfactory sensory acceptability, although the results were better for guava. The fruit by-products can be used as raw materials for the development of alternative kombucha beverages.

**Novelty and scientific contribution:**

As consumers are increasingly selective in their food choices, the development of food products with high nutritional value has increased significantly in recent years. New types of fermentable beverages such as kombucha - using tropical fruit by-products to enhance their chemical composition, sensory properties and nutritional value - have created new opportunities for beverage consumption and offer greater health benefits than the traditional version, where only *Camellia sinensis* is used. The promotion of these co-products and their respective beverages is an excellent opportunity for sustainability and their commercialisation.

## INTRODUCTION

The large amount of by-products generated by agro-industrial processing is one of the challenges of the 21st century (*1*). Worldwide, 1.3 billion tonnes of by-products are generated annually, including processed by-products and waste from the production chain. According to FAO (*2*), most of these come from fruit and vegetables, which account for up to 50 % of production, mainly in the processing and postharvest stages (*3*).

Brazil is a country that produces a wide range of agro-industrial by-products. Due to poor management, this practice can have serious environmental impacts. Acerola, guava and tamarind are perishable products that are sensitive to exogenous factors. During production, high rates of postharvest losses occur, generating a large amount of by-products and non-recyclable waste. It is estimated that waste after harvest and production of juices are very high, reaching losses of 10 to 50 % (*4*).

The search for viable and economical use of agro-industrial by-products is necessary if the food industry wants to be more sustainable. It also has an impact on the reduction of raw material waste and production costs (*5*). Many food formulations have been investigated that aim to use artisanal or industrial by-products, leading to an expansion of the plant-based food production.

Fruit by-products can have a high nutritional content, often higher than that of their edible parts. Furthermore, these by-products may also contain bioactive compounds with a higher antioxidant capacity than the pulp (*6*). In addition, agro-industrial by-products contain many fermentable sugars and nutrients from which microorganisms can produce various substances of industrial importance that can be used for the development of various products, such as kombucha (*7*).

Kombucha is a slightly sweet and acidic drink generally produced by fermenting black or green tea (*Camellia sinensis*) using sugar and a cellulose biofilm containing a symbiotic culture of bacteria and yeasts known as SCOBY (*8*).

The literature has already described the use of other products for the production of kombucha. Examples include herbal infusions, wax mallow flowers, coffee, oak leaves, eucalyptus, bay leaves, fruit juices, milk and soya products, which have proven to be good alternatives to black or green tea (*9*, *10*).

In addition, depending on the raw material used to produce kombucha, the final product can have improved chemical composition, sensory and biological properties, which open up new possibilities for beverage production and potentially offers products with more health benefits for the consumers (*10*).

With this in mind, the study presented here aims to develop kombucha analogues using the agro-industrial by-products of fruit pulp processing. In addition to offering a new product for the consumers, the preparation of the beverage is associated with the use of fruit by-products and the reduction of their environmental impact, minimising their improper disposal. Formulations were made using by-products that were preselected based on their antioxidant activity, and their fermentation kinetics, physicochemical and sensory properties, and organic acid profile were investigated.

## MATERIALS AND METHODS

### Raw material

By-products of six tropical fruits - acerola (*Malpighia emarginata*), guava (*Psidium guajava*), tamarind (*Tamarindus indica*), passion fruit (*Passiflora edulis*), mombin (*Spondias mombin*) and pineapple (*Ananas comosus*) – were used as raw materials (supplied by Nossa Fruta, a fruit pulp company in Eusébio, Brazil) for the production of fermented beverages, together with sugar (União®, São Paulo, Brazil) and potable water (Naturagua®, Fortaleza, Brazil). The samples were registered in the National System for the Management of Genetic Heritage and Associated Traditional Knowledge (SISGEN) under accession number AA72205 through the Federal University of Ceará, Fortaleza, Brazil.

For the fermentation process, both the symbiotic culture of bacteria and yeasts (SCOBY) and the liquid from the end of the kombucha fermentation test (prepared beforehand) were used as starter culture (provided by our research group, Fortaleza, Brazil). After fermentation, the fruit pulp (supplied by a fruit pulp Nossa Fruta), which corresponds to the by-product of each formulation, was used in the flavouring stage.

### Selection of by-products

In order to select the by-products with the best properties for the production of fermented beverages, antioxidant activity was first investigated using the 2,2'-azino-bis(3-ethylbenzothiazoline-6-sulphonic acid) radical scavenging (ABTS˙^+^; Sigma-Aldrich, Merck, MO, USA) method as described by Re *et al.* (*11*) and adapted by Rufino *et al.* (*12*). Different concentrations of the by-products were used and the beverage properties depended on each fruit. An aliquot of 30 μL of each dilution (selected according to the calibration curve) reacted with 3 mL of the ABTS˙^+^ solution in the dark. The absorbance values of the reaction mixture were measured after 6 min in a spectrophotometer (Kasvi, São José dos Pinhais, Brazil) at 734 nm. A standard curve between 100 and 1500 μM of Trolox (6-hydroxy-2,5,7,8-tetramethylchroman-2-carboxylic acid; Sigma-Aldrich, Merck) was used as a reference. The measured antioxidant capacity of the samples was expressed as Trolox equivalents (μM/g). Based on these results, three by-products were selected to develop the formulations.

### Kombucha formulations

**Fig. S1:**
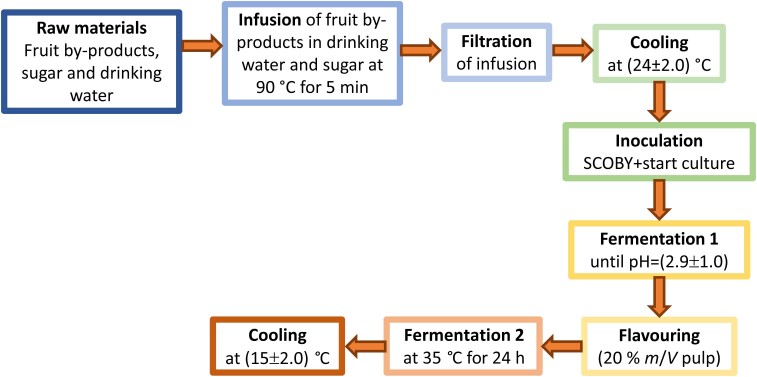
Flowchart of the process for the development of fermented beverages. SCOBY=symbiotic culture of bacteria and yeasts

The preparation of all formulations (Fig. S1) started with the infusion phase (at (90±2) °C for 5 min) using drinking water (1000 mL), the specific fruit by-products (10 % *m*/*V*) and 10 % sugar. After the infusion phase, the samples were filtered in felt tissue to remove solid residues. Then the resulting liquid was cooled to (24±2) °C and *φ*(kombucha)=15 % and 20 % (*m*/*V*) SCOBY were added, starting fermentation process 1 (F1) in the presence of oxygen. The SCOBY used for all formulations was from the same initial fermentation, and the same amount of this culture was used for all formulations.

As each tested fruit by-product could have a different fermentation kinetics, the fermentation time of each formulation was determined as a function of the pH (model 3505; Jenway, Chelmsford, UK) that was established for all formulations (2.9±1.0) according to the Brazilian Normative Instruction No. 41 (*13*), which determines a minimum and maximum pH of 2.5 and 4.2 (*14*, *15*) for kombucha, respectively.

The second fermentation (F2) was carried out to impart gas and flavour to the beverage, adding 20 % (*m*/*V*) of pulp to each formulation. The fermented beverages were then transferred to PET bottles and stored at room temperature (25 °C) for 24 h. Each formulation was prepared in triplicates to ensure a more reliable result. Once the flavouring phase was completed, the formulations were refrigerated at (12±2) °C to slow down the fermentation process. A total of three formulations were prepared after the selection of the by-products: acerola by-product (FBA), guava by-product (FBG) and tamarind by-product (FBT).

### Fermentation kinetics

The fermentation kinetics of the formulations was evaluated by pH, titratable acidity and total soluble solids content during the first (F1) and the second (F2) fermentation, the latter also known as flavouring fermentation. During F1, kinetics was measured at 0, 48, 72, 96 and 168 h and during F2 at 0 and 24 h.

The soluble solid content was measured by direct reading of the samples, where an aliquot of each sample was added to the prism (*14*). These direct readings were done with a portable refractometer (ASKO, São Leopoldo, Brazil), model RT 32.

The pH was determined with potentiometric method using a digital pH metre (model 3505; Jenway) calibrated with pH buffer solutions of 4.0 and 7.0, following the procedure described by Adolfo Lutz Institute (*14*).

Titratable acidity (TA) was determined by the titrimetric method using 0.1 M NaOH. The results were expressed as percentage of acetic acid (*14*).

### Microbiological analyses

Total coliforms, thermotolerant coliforms, *Escherichia coli* and aerobic mesophilic bacteria were counted fusing the rapid analysis method known as Compact Dry (Nissui Pharmaceutical CO, Taito-ku, Tokyo), certified by the AOAC International (*15*). *Salmonella* sp. was analysed according to the Bacteriological Analytical Manual (*16*) to evaluate the safety of the developed product. The samples were subjected to pre-enrichment (lactose broth; Kasvi), followed by selective enrichment (tetrathionate broth and Rappaport-Vassiliadis broth; Kasvi) and plating media for *Salmonella* (xylose lysine deoxycholate agar, Hektoen agar and bismuth sulphite agar; Kasvi).

### Determination of organic acids

All samples were injected into HPLC chromatograph LC 10AvP (Shimadzu, Tokyo, Japan) to determine the organic acid content. Before injection, they were filtered through polyvinylidene difluoride (PVDF) filters of 0.45 and 0.22 μm. All mobile phases passed through a 0.22 μm cellulose acetate filter.

A 214 nm UV–Vis detector (SPD-M10AVP; Shimadzu) and LiChrospher® 100 RP-18 column (4.6 mm×250 mm; 5 μm) were used to determine the organic acid content. The 0.2 M KH_2_PO_4_ (pH=2.4) was used as the mobile phase and the flow rate was 0.8 mL/min (*17*). The profiles of glucuronic, lactic, acetic, citric and ascorbic acid were determined based on standard curves previously determined for each substance (Table S1). For all analyses, the column oven remained at 40 °C (CTO-10 AS VP; Shimadzu) and the injection loop was 20 μL. For each organic acid, the limit of detection (LOD), *i.e.* the lowest concentration of the analyte that can be detected, and the limit of quantification (LOQ), *i.e.* the lowest concentration of the analyte that can be measured, were determined.

### Sensory analysis

The study was submitted to the Ethics Committee (Animal Use Ethics Committee of the Federal University of Ceará, Brazil) and approved with the number 4.729.905. Sensory properties of the selected and optimised formulations were evaluated using an adapted method due to the pandemic caused by COVID-19 and the requirement for social isolation. Sensory analysis was performed on 23 September 2021. Thus, for this study, the check-all-that-apply (CATA), rate-all-that-apply (RATA) and acceptance tests were carried out at the tasters' homes. The preventive measures according to the Brazilian National Health Surveillance Agency (*18*) were followed in the production and delivery of the formulations, such as the use of gloves and masks.

The acerola, guava and tamarind by-products were delivered to each taster's home in PET bottles containing approx. 100 mL of product, along with a letter containing instructions and the link and QR code to access the form. The tasters were asked to drink mineral water at room temperature between samples to cleanse the palate (*14*).

The tests were conducted with a group of 60 untrained panellists who were healthy, non-smokers and selected based on their consumption of fermented products and acerola juice, as well as their previous experience with sensory tests. The group consisted of 36 women and 24 men aged between 18 and 65, with more than 85 % of them being younger than 50.

The samples were evaluated in 100-mL plastic cups. Acceptance test forms were available with scores ranging from 1 (I disliked it a lot) to 9 (I liked it a lot) to check the participants’ level of acceptance of appearance, aroma, taste and overall acceptability (*19*, *20*). In the CATA test (*21*), tasters had to select among 20 descriptive terms related to appearance, taste and aroma those that best represented the type of the tested product (listed in Table 1). In the RATA test, the tasters had to rate the applicability of the terms to the samples on a five-point scale, with one being very little and five being very much. The terms used in the RATA test were appearance, aroma and flavour, and the attributes used for each term corresponded to the characteristics present in each formulation.

**Table 1:** Multiple comparisons of check-all-that-apply (CATA) and rate-all-that-apply (RATA) test results for each attribute in all samples using the McNemar (Bonferroni) procedure and Cochran’s Q test to compare each attribute in kombucha formulations fermented with fruit by-products

	CATA					RATA			
Attribute	FBA	FBG	FBT	p-value		FBA	FBT	FBG	p-value
Bright	(0.7±0.4)^a^	(0.7±0.4)^a^	(0.7±0.5)^a^	0.5		(1.9±1.3)^a^	(1.7±1.3)^a^	(1.9±1.3)^a^	0.6
Translucent (clear)	(0.6±0.4)^a^	(0.6±0.4)^a^	(0.6±0.4)^a^	0.7		(1.7±1.5) ^a^	(1.4±1.3)^a^	(1.6±1.5)^a^	0.6
Homogeneous	(0.8±0.3)^a^	(0.8±0.4)^a^	(0.7±0.4)^a^	0.6		(2.3±1.3)^a^	(2.1±1.4)^a^	(2.4±1.5)^a^	0.6
Sedimented	(0.7±0.4)^a^	(0.6±0.4)^a^	(0.7±0.4)^a^	0.5		(1.8±1.4)^a^	(2.0±1.6)^a^	(1.7±1.5)^a^	0.5
Presence of bubbles	(0.8±0.3)^a^	(0.8±0.4)^a^	(0.8±0.4)^a^	0.5		(2.3±1.4)^a^	(2.2±1.6)^a^	(2.5±1.7)^a^	0.4
Sweet scent	(0.7±0.4)^a^	(0.8±0.3)^a^	(0.7±0.4)^a^	0.0		(1.4±1.1)^b^	(1.6±1.2)^b^	(2.6±1.3)^a^	<0.0001
Citrus scent	(0.8±0.3)^a^	(0.8±0.3)^a^	(0.8±0.3)^a^	0.8		(2.6±1.5)^a^	(2.2±1.4)^a^	(2.1±1.4)^a^	0.1
Acid flavour	(0.7±0.4)^a^	(0.7±0.4)^a^	(0.8±0.4)^a^	0.5		(2.2±1.6)^a^	(2.2±1.5)^a^	(1.7±1.4)^a^	0.1
Vinegar scent	(0.6±0.4)^a^	(0.6±0.4)^a^	(0.7±0.4)^a^	0.1		(1.6±1.4)^a^	(1.9±1.5)^a^	(1.4±1.3)^a^	0.1
Fermented flavour	(0.7±0.4)^a^	(0.7±0.4)^a^	(0.7±0.4)^a^	0.8		(1.9±1.4)^a^	(1.9±1.4)^a^	(1.6±1.3)^a^	0.2
Acid taste	(0.8±0.3)^a^	(0.9±0.3)^a^	(0.8±0.3)^a^	0.0		(2.8±1.7)^a^	(2.8±1.4)^a^	(2.5±1.4)^a^	0.6
Sweet taste	(0.7±0.4)^a^	(0.7±0.4)^a^	(0.7±0.4)^a^	0.6		(1.6±1.2)^b^	(1.6±1.3)^b^	(2.1±1.4)^a^	0.0
Salty taste	(0.5±0.4)^a^	(0.5±0.4)^a^	(0.6±0.4)^a^	0.5		(1.0±1.2)^a^	(1.0±1.0)^a^	(0.9±1.0)^a^	0.9
Bitter taste	(0.6±0.4)^a^	(0.6±0.4)^a^	(0.6±0.4)^a^	0.1		(1.4±1.5)^a^	(1.5±1.5)^a^	(1.2±1.3)^a^	0.5
Citrus flavour	(0.9±0.2)^a^	(0.8±0.3)^a^	(0.8±0.3)^a^	0.0		(3.1±1.3)^a^	(2.5±1.3)^b^	(2.4±1.3)^b^	0.0
Vinegar flavour	(0.7±0.4)^b^	(0.6±0.4)^a^	(0.7±0.4)^ab^	0.0		(1.8±1.5)^a^	(1.6±1.4)^a^	(1.5±1.5)^a^	0.6
Fermented flavour	(0.8±0.4)^a^	(0.7±0.4)^a^	(0.7±0.4)^a^	0.6		(2.1±1.5)^a^	(1.9±1.4)^a^	(1.8±1.4)^a^	0.4
Astringent sensation	(0.5±0.4)^a^	(0.5±0.5)^a^	(0.5±0.5)^a^	0.2		(1.5±1.6)^a^	(1.4±1.5)^a^	(1.3±1.4)^a^	0.7
Spicy sensation	(0.5±0.5)^a^	(0.5±0.5)^a^	(0.5±0.5)^a^	0.7		(0.9±1.0)^a^	(1.0±1.2)^a^	(1.1±1.3)^a^	0.6
Frizzling sensation	(0.7±0.4)^a^	(0.7±0.4)^a^	(0.7±0.4)^a^	0.4		(1.6±1.4)^a^	(1.7±1.3)^a^	(1.7±1.3)^a^	0.9

Mean values with the same letters in the same row do not differ at the 5 % significance level for the CATA and RATA analyses, separately. FBA, FBG and FBT=formulation with acerola, guava and tamarind by-product, respectively

Purchase intention was also evaluated using a structured five-point scale, with five representing ’I would certainly buy it’ and one representing ’I would certainly not buy it’ (*20*).

### Statistical analysis

Statistical analysis was performed using analysis of variance (ANOVA) and Tukey’s test (p=0.05), evaluating significant differences between samples subjected to the same analysis. The results of fermentation kinetics were evaluated by regression analysis using Systat software v. 13.2 (*22*). The data obtained from the sensory analysis were first analysed in terms of variance (ANOVA) and then the mean values of the hedonic values were subjected to the Tukey’s test at 5 % probability. The Cochran Q test was used to compare the frequency means of each CATA attribute. The results were also analysed by principal component analysis (PCA) and presented in two-dimensional graphs. Penalty analysis was performed on the data obtained by CATA to determine whether a present attribute caused a lower or higher preference or did not affect the preference of the samples (*23*). The results were analysed to assess which attributes should be present (’must have’) or absent (’must not have’) (*24*). All tests were performed with the Assistat software v. 7.7 beta (*25*) except for the sensory programme, which was performed with the XLSTAT software v. 4.5 (*26*).

## RESULTS AND DISCUSSION

### Antioxidant activity of fruit by-products

Based on the antioxidant activity values obtained, expressed as Trolox equivalent antioxidant capacity (TEAC), three fruit by-products were selected to be used for the development of the fermented beverages: acerola (44.6±0.4), tamarind (28.3±0.3) and guava (2.06±0.05) μmol/g. The guava by-product replaced the mombin by-product (3rd place, (5.70±0.02) μmol/g) as raw material due to the off-season of the mombin fruit. Passion fruit and pineapple had lower values of antioxidant activity ((1.30±0.03) and (1.20±0.01) μmol/g, respectively. Therefore, they were not selected for the preparation of beverages.

The evaluation and consumption of compounds with antioxidant capacity have attracted the interest of researchers and consumers as they are associated with the reduction of degenerative diseases caused by free radicals. Antioxidant capacity is attributed to the ability of the sample to quench free radicals by donating either hydrogen atoms or electrons. Therefore, they can prevent the harmful effects of oxidation and provide several health benefits when included in the human diet (*27*, *28*). These benefits are associated with the presence of ascorbic acid, anthocyanins, carotenoids, phenolic compounds and other antioxidants that are easily found in various fruits and vegetables (*29*).

Silva *et al.* (*6*) evaluated bioactive compounds from tropical fruit residues and found high amounts of total phenolic compounds in acerola, guava and mango residues. The content of phenolic compounds could be related to the antioxidant activity of the by-product, although the results obtained in this study are consistent with those reported by the authors.

### pH, titratable acidity and total soluble solids during kombucha fermentation

The pH is an important parameter in fermentation processes, as it reduces the growth of pathogenic microorganisms (pH*<*4.5) and prevents structural changes in antioxidant compounds (*30*).

**Fig. 1:**
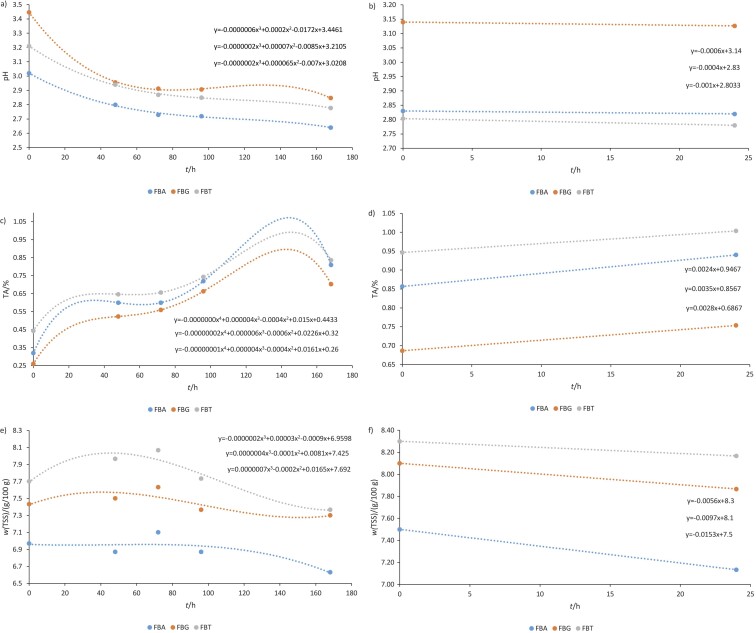
The effect of fermentation time of formulations with acerola, guava or tamarind by-products (FBA, FBG and FBT, respectively) on the average of: a) and b) pH during fermentation 1 and 2, c) and d) titratable acidity (TA) as acetic acid during fermentation 1 and 2, and e) and f) total soluble solids (TSS) during fermentation 1 and 2, respectively

During the first fermentation (Fig. 1a), a decrease in pH over time was observed. The initial pH of the acerola by-product was 3.02; after 48 h it decreased to 2, and at the end of F1 it changed to 2.64. Similar results were observed for the guava by-product, where the initial pH was 3.45 and the final pH was 2.85. The pH of tamarind by-product decreased from 3.21 to 2.78 at the end of F1.

After F1, the pH was measured during the flavouring stage (Fig. 1b), which lasted 24 h, and values were measured at 0 and 24 h, when a slight decrease in pH was observed in all formulations. It can be seen that F1 was responsible for a more significant decrease in pH.

In a previous study, the pH of kombucha fermented with rice and barley decreased from the sixth to the eighth day of fermentation (*31*), which is in alignment with the results of the present study. Similar results were found by Leonarski *et al.* (*30*), who prepared kombucha beverages using an acerola by-product. The initial pH of beverages prepared with 1, 3 and 5 % acerola by-product was 3.24, 3.34 and 3.27, respectively, with all samples showing similar behaviour, including a decrease in pH until the 12th day of cultivation. After 15 days of fermentation, a pH of 2.49, 2.54 and 2.58, respectively, was observed, which is very close to the values at the end of F2 in the present study (pH=2.82, 3.12 and 2.78 for FBA, FBG and FBT, respectively; Fig. 1b).

This decrease observed in all formulations can be attributed to the production of organic acids during fermentation, which causes the reduction of the pH of kombucha, reducing the number of possible pathogens and generating a safe drink for consumption, despite being of microbial origin (*32*). According to Rodrigues *et al.* (*33*), a final pH of 2.5 signals the end of the fermentation process, which is close to that observed at the end of F1 in our study (Fig. 1c and Fig. 1d).

Fig. 1c and Fig 1d show a gradual increase in acidity in all formulations during fermentations 1 and 2. The FBA started with an acidity of 0.32 %, evolving to 0.94 % at the end of F2. On the other hand, FBG had an initial acidity of 0.26 % and a final acidity of 0.75 %. The FBT was the formulation with the highest initial and final acidity (0.44 and 1 %, respectively). Finally, the FBG was the formulation that had the lowest acidity.

Hibiscus-based kombucha (*33*) had 0.18 % titratable acidity, which is lower than the values found in the present study. This can be explained by the difference in fermentation time and the composition of SCOBY. Tanticharakunsiri *et al.* (*34*) prepared kombucha with oolong tea fermented for seven days and found a titratable acidity of approx. 0.6 %, close to what was found at certain times for some formulations in the present study, such as the FBA, with value of 0.6 % at 48 and 72 h (Fig. 1c).

A significant increase in titratable acidity during the fermentation process is expected due to the production of characteristic acids formed as a result of the metabolism of acetic acid bacteria. However, the oscillations observed in this experiment may be due to the rapid volatilization of acetic acid that may have occurred during sample collection or even during the fermentation period due to the portage of the tissue used for nozzle coverage, which may have facilitated the volatilization of acids. A total titratable acidity content between 0.40 and 0.45 % was reported as indicative of the completion of the fermentation process (*35*).

The production of acids during the fermentation process justifies the variation of total soluble solids since the acetic acid bacteria present in SCOBY consume sugars, converting them into organic acids (*36*).

At the start of F1, FBA had a total solid content of 6.97 mg/100 g, and at the end 6.63 mg/100 g (Fig. 1e), while at the start of F2, it had significantly higher total solid content (7.50 mg/100 g), which was reduced to 7.13 mg/100 g after 24 h. At the start of F1, FBG had the total solid content of 7.43 mg/100 g and at the end it had 7.30 mg/100 g. It increased at the beginning of F2 (8.10 mg/100 g) and was reduced after 24 h (7.87 mg/100 g). The same observations were perceived for FBT, at the beginning of F1 it had a total solid content of 7.70 mg/100 g and at the end 7.37 mg/100 g, which increased at the start of F2 (8.30 mg/100 g) and reduced to 8.17 mg/100 g at the end of F2 (Fig. 1f).

The soluble solid content was reduced in all formulations during both fermentations (F1 and F2). Their increase at the start of F2 (flavouring stage) can be attributed to the addition of fruit pulp during this stage.

Filho *et al.* (*37*) developed fermented drinks with kombucha and kefir and found a reduction in the total soluble solid content with fermentation time, a result similar to that obtained in the present study.

### Antioxidant activity and the microbiological safety of kombucha beverages

It is possible to notice reduction in values associated with both dilutions used for beverage preparation and the temperature applied during production. Moreover, a significant difference between one formulation and another may be related not only to the fact that different fruit co-products were used, but also to the climatic conditions and soil composition from which the fruits were harvested (*38*).

The kombuchas fermented with green tea (*39*) had an antioxidant activity expressed as Trolox equivalents of 11.35 to 11.50 μmol/g, which is lower than the ones found in the present study for FBT ((20.0±0.8) μmol/g, while kombuchas fermented with black tea (*40*) had an antioxidant activity ranging from (0.4±0.1) to (9.6±0.3) μmol/g. Antioxidant activity demonstrated in kombucha formulations is of great economic and nutritional importance, as they are beverages made from fruit by-products that still have considerable antioxidant activity.

The developed formulations are safe for consumption from the microbiological point of view, with no *Salmonella* detected in 25 mL and counts of total and thermotolerant coliforms, *E. coli* and aerobic mesophiles lower than 1 CFU/mL.

Içen *et al.* (*41*) evaluated the antimicrobial potential of kombucha against Gram positive and Gram negative bacteria and found that it has antimicrobial properties towards a wide variety of pathogens (*Salmonella* sp., *L. monocytogenes*, *Staphylococcus* spp., *C. albicans*, among others), which is confirmed by the absence of *Salmonella* and *E. coli* in tested samples in this study.

Furthermore, the use of good manufacturing practices can explain the excellent microbiological quality of the formulations, in addition to the physicochemical and microbial characteristics associated with the fermentation process, such as the pH and presumably high counts of acetic acid bacteria and yeasts, which inhibit the growth of undesirable microorganisms (*42*).

### Organic acids present in kombucha formulations

The three main functional acids associated with kombucha are gluconic acid, d-saccharic acid-1,4-lactone (DSL) and glucuronic acid (*43*). Glucuronic acid is one of the most important acids in the organism. It is formed in kombucha as a result of glucose oxidation by microorganisms, and is recognised as one of the natural substances with the highest detoxifying potential. It can bind to toxins, increasing their water-solubility and, consequently, making them easier to eliminate by urine (*44*). Bacteria such as *Acetobacter*, *Gluconobacter* and *Komagataeibacter* spp. (*45*) use sugar and ethanol (*46*) to produce gluconic acid, therefore, its presence in tested formulations is possibly linked to the growth of these bacteria.

Glucuronic acid was the main organic acid detected in kombucha samples (Table 2), ranging from 8.0 (FBG) to 14.6 mg/100 mL (FBA). The second organic acid with the highest presence was the acetic acid, which ranged from 5.0 (FBT) to 14.67 mg/100 mL (FBA). Other research (*37*) involving the analysis of acids in kombucha also identified and quantified the presence of glucuronic acid in the formulations, reinforcing that it is an acid generally present in kombuchas, even with changes in fermentative substrates.

**Table 2:** Organic acid quantification in formulations of kombucha fermented with fruit by-products, and limits of detection and quantification of HPLC measurement for standard substances used

	*γ*(organic acid)/(mg/mL)				
Formulation	Glucuronic	Ascorbic	Lactic	Acetic	Citric
FBA	(14.6±0.2)^a^	(3.28±0.05)^a^	(3.16±0.02)^a^	(14.67±0.08)^a^	(0.00±0.00)^b^
FBG	(8.0±0.2)^c^	(0.14±0.00)^b^	(0.00±0.00)^b^	(7.27±0.06)^b^	(2.60±0.05)^a^
FBT	(11.7±0.4)^b^	(0.00±0.00)^c^	(0.00±0.00)^b^	(5.0±0.2)^c^	(0.00±0.00)^b^
LOD/(mg/mL)	0.001	0.001	0.01	0.01	0.000001
LOQ/(mg/mL)	0.005	0.001	0.03	0.05	0.000005

FBA, FBG and FBT=formulation with acerola, guava and tamarind by-product, respectively. The mean value±standard deviation (*N*=3) followed by equal letters in the same column do not differ from each other according to Tukey’s test (p>0.05). LOD=limit of detection, LOQ=limit of quantification

The formulations that had a lower pH at the end of F2 were FBT and FBA with pH=2.78 and 2.82, respectively (Fig. 1b). We know that a lower pH is related to a higher production of organic acids, such as glucuronic acid, a fact found in the present study, where FBA was the formulation with the highest amount of glucuronic and acetic acids.

Another important fact to be highlighted is the variation of total soluble solids, as the acetic bactera in SCOBY consume sugar, converting it into organic acids. Therefore, the higher the amount of total soluble solids in a formulation, the lower the amount of acetic acid. This was observed in the present study, where the formulation that obtained the lowest amount of total soluble solids was the FBA (7.13 %; Fig. 1f), also being the formulation with the highest acetic acid production (14.67 mg/100 mL). On the other hand, FBT – the formulation with the lowest acetic acid production (5.0 mg/100 mL) – had a higher amount of total soluble solids (8.17 mg/100 g).

Black tea kombucha contains 3.23 mg/100 mL of glucuronic acid (*47*), a value lower than those found in all beverages in the present study (Table 2). The concentration of acetic acid in the present study was notably higher (9.18 mg/100 mL) than in black tea samples. This suggests that factors such as fermentation, type of raw material or storage conditions may have influenced the increased levels. The findings highlight significant differences in acetic acid content between the teas, emphasissing the need to further explore what drives these variations. The main metabolic characteristic of lactic acid bacteria is known as the ’primary acidification process’, referring to the carbohydrate consumption that generates acid. This action is crucial for rapid pH reduction, protecting food against decaying and pathogenic microorganisms.

Due to the fact that yeasts and lactic acid bacteria use sugars as a fermentation substrate, the predominance of lactic acid concentration is not unexpected. Simultaneously, sugar is still available in the medium, something that may have occurred in FBA, in which lactic acid concentration was 3.16 mg/100 mL.

Citric acid was found only in FBG beverage (2.60 mg/mL; Table 2). Ascorbic acid was present in FBG and FBA, ranging from 0.14 to 3.28 mg/mL, respectively. The low concentration of ascorbic acid may be associated with temperature variation, as ascorbic acid is susceptible to high temperatures, generally above 40 °C (*48*). Additionally, it should be considered that an industrial residue can be pressed repeatedly in order to obtain a higher pulp yield, and this single operation can influence the final bioactive content compared to the pulp.

According to the Recommended Dietary Allowances (*49*), the minimum amount of ascorbic acid per day required for an adult is 60 mg. This indicates that the 300 mL intake of acerola kombucha would supply approx. 16.4 % of this recommendation.

### Sensory evaluation of kombucha beverage

The majority of the participants in the sensory analysis were female (60 %). Regarding age, 37 % were between 18 and 25 years old, 32 % were from 26 to 35, 18 % were from 36 to 50, 8 % were between 51 and 65 years old, and only 5 % were under 18.

Cochran's Q test result (Table 1) indicated the terms most cited by the participants. The four significant descriptors at the level of 5 % were sweet aroma, acidic taste, citric taste, and vinegar flavour, which were used to perform CATA.

Mendonça *et al.* (*50*) evaluated kombucha made from unconventional parts of *Hibiscus sabdariffa* L. and the samples were characterised by the sensory panel as vinegar aroma, vinegar flavour, sour taste or more viscous. Thus, similar characteristics were obtained when comparing the samples evaluated in the present study regarding vinegar flavour.

**Fig. 2:**
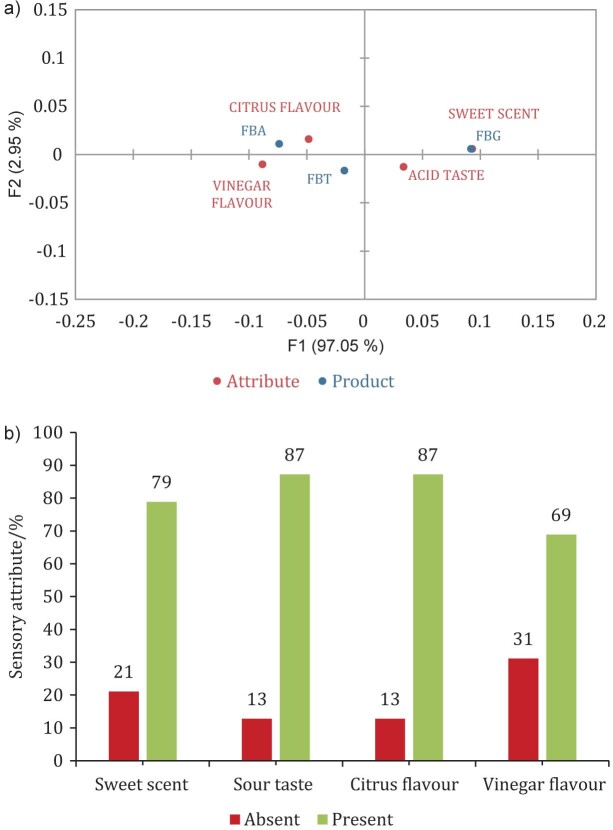
a) Analysis of the main components from check-all-that-apply (CATA) data in kombucha formulations fermented with fruit by-products, and b) histogram showing the percentages of sensory judges who identified (present) and did not identify (absent) significant sensory attributes in the CATA test at a 5 % significance level. FBA, FBG and FBT=formulation with acerola, guava and tamarind by-product, respectively

Fig. 2 shows the correlation between formulations and sensory attributes. It can be verified that the attributes most related to the FBA formulation were fermented flavour, vinegar flavour, citrus aroma, salty taste and astringent sensation.

Treviso *et al.* (*51*) developed kombucha separately from the yerba mate infusion and obtained a product sensorially characterised by a fermented flavour, a characteristic similar to that observed in this research.

FBA, FBG and FBT formulations have different sensory characteristics (as shown in Fig. 2), which is expected since different fruit by-products were used, and other factors were involved in the fermentation. FBA is correlated with the attribute citrus flavour, FBT with vinegar flavour and FBG with sweet taste. Although the acid taste is more related to FBG, it is also closer to the overall evaluation, indicating its preference among the samples evaluated by the tasters.

Fig. 2 shows the percentage of presence and absence of attributes with a significance of 5 %, in which sour taste and citrus taste were the attributes with the highest perception (87 %) among the tasters. After the sweet aroma attributes, with 79 %, the vinegar taste was observed at 69 %. Table 1 shows that the sweet aroma, sweet taste, and citrus flavour differed between the samples at 5 % significance level in the RATA test.

The terms bright, translucent (clear), homogeneous, sedimented, presence of bubbles, citrus aroma, acidic aroma, vinegar aroma, fermented aroma, acidic taste, sweet taste, salty taste, bitter taste, vinegar flavour, fermented flavour, astringent sensation, spicy sensation, and crimping sensation did not differ throughout the number of results among the samples evaluated.

Regarding the FBT formulation, the appearance attribute obtained a score corresponding to ‘liked it a little’, with an average of 6.0, differing significantly (p≤0.05) from the FBG and FBA formulations (Table 3), which were evaluated with averages of 7.3 and 6.8, respectively, both between ‘liked it’ and ‘liked it a little’.

**Table 3:** Mean values for the acceptance test of FBA, FBG and FBT formulations for the parameters of appearance, aroma, flavour, overall assessment and purchase intention of the tasters

Beverage	Appearance	Aroma	Flavour	Overall assessment	Purchase intention
FBG	(7.3±1.3)^a^	(7.2±1.4)^a^	(7.0±1.5)^a^	(7.0±1.5)^a^	(3.7±1.0)^a^
FBT	(6.0±2.0)^b^	(6.0±1.9)^b^	(6.2±1.9)^b^	(6.2±2.0)^b^	(3.3±1.0)^b^
FBA	(6.8±1.6)^a^	(5.6±2.1)^b^	(6.1±18)^b^	(6.0±1.9)^b^	(3.2±1.1)^b^
Pr>F (model)	0.000	<0.0001	0.009	0.012	0.009

Mean values with the same letters in the same row do not differ at the 5 % significance level. FBA, FBG and FBT=formulation with acerola, guava and tamarind by-product, respectively

Regarding the aroma attribute, the FBG sample was observed to differ significantly (p≤0.05) from the FBT and FBA formulations, obtaining an average of 7.2 between ‘liked it’ and ‘liked it a lot’. The FBT and FBA formulations were rated with averages of 6.0 and 5.6, respectively, between the terms ‘neither liked nor disliked it’ and ‘liked it a little’.

The sample that received a better overall evaluation was the FBG (7.0), being close to the ‘liked it’ attribute and differing significantly (p≤0.05) from the FBT and FBA formulations, which received averages of 6.2 and 6.0, respectively, both characterised by the ‘I liked it a little’ attribute.

Formulations with different fermentative substrates will have their own acceptability characteristics, making it necessary to carry out sensory tests to confirm that the product has the potential to be accepted by potential consumers. Currently, studies involving the production of kombuchas developed with alternative substrates have demonstrated the potential associated with the acceptance of these products (*52*, *53*).

The tasters attributed the FBG formulation a higher score for the purchase intention, presenting an average of 3.7, being close to the ‘I would probably buy it’ attribute. It differs significantly (p≤0.05) from the FBT and FBA formulations, which averaged 3.3 and 3.2, respectively. Thus, both were classified with the ‘maybe I would buy it, maybe not’ attribute. Therefore, it is verified that, among the formulations studied, the one that obtained the highest score in all sensory parameters was the FBG, which may be associated with consumer preference for guava fruit.

## CONCLUSIONS

Considering the chemical and physical parameters, the fermented acerola, guava and tamarind beverages have achieved satisfactory results. Taking into account the values established by current legislation, they are considered safe for human consumption from a microbiological point of view. During the evaluation of fermentation kinetics, it was observed that the pH and soluble solid content decreased with increasing acidity. Among all beverages, those obtained using acerola by-product had the highest antioxidant potential.

An excellent amount of organic acids was found in all formulations. The beverage produced from acerola by-product had the highest concentrations of glucuronic, ascorbic, lactic and acetic acids. The only formulation in which citric acid was quantified was guava by-product.

The formulation that received the highest acceptance score among the tasters was the one with the guava by-product, followed by the formulation with the tamarind by-product and the one with the acerola by-product. All samples were considered acceptable by the panellists. The importance of this work extends to the associated benefits, as it offers new food alternatives with high nutritional content and fully utilises fruit by-products.

## Appendix

**Table S1:** HPLC measurements of standard organic acids

Organic acid	*t*_R_/min	Calibration curve	Linear correlation	*γ*(linear range)/(mg/mL)
Acetic	5.77	y=6044469x-30333	R^2^=0.9999	0.1–25.0
Glucuronic	2.67	y=543029x-22176	R^2^=0.9999	0.5–20.0
Lactic	5.11	y=758060x-24574	R^2^=0.9995	0.0425–4.24
Citric	6.42	y=10^6^x-26252	R^2^=0.9978	0.1–3.0
Ascorbic	4.32	y=1.65·10^7^x-1.74·10^5^	R^2^=0.9972	0.1–5.0
